# High- and Low-Calorie Food Cues, Visual Attention, and Subclinical Eating Pathology in University Students Assessed by Eye Tracking

**DOI:** 10.3390/nu18172899

**Published:** 2026-09-03

**Authors:** Csongor István Szepesi, Viktor Rekenyi, Nóra Horváth, Róbert László Nagy, Mihály Soós, Anita Szemán-Nagy, Zoltán Kondé, Győző Kurucz, Luo Xiaonuo, László Róbert Kolozsvári

**Affiliations:** 1 Doctoral School of Health Sciences, University of Debrecen, 4032 Debrecen, Hungary; szepesi.csongor@med.unideb.hu (C.I.S.);; 2 Department of Family and Occupational Medicine, Faculty of Medicine, University of Debrecen, 4032 Debrecen, Hungary; 3 Faculty of Economics and Business, Institute of Marketing and Commerce, University of Debrecen, 4032 Debrecen, Hungary; 4 Institute of Psychology, Department of Personality and Clinical Psychology, University of Debrecen, 4032 Debrecen, Hungary; 5 Institute of Psychology, Department of General Psychology, University of Debrecen, 4032 Debrecen, Hungary; 6 Institute of Psychology, Department of Social and Work Psychology, University of Debrecen, 4032 Debrecen, Hungary

**Keywords:** eating disorders, eye-tracking, attentional bias, EAT-26, subclinical eating pathology, caloric density, visual attention, oculomotor metrics

## Abstract

**Background/Objectives**: Eating disorders are increasingly conceptualized as information-processing disorders characterized by attentional biases toward food-related stimuli. However, whether such biases are already detectable in subclinical, non-treatment-seeking populations remains unclear. This study examined whether subclinical eating pathology, assessed with the Eating Attitudes Test-26 (EAT-26), is associated with distinct patterns of visual attention toward high- and low-calorie food images in university students. **Methods**: In this cross-sectional observational study featuring an experimental eye-tracking task, 89 university students completed the EAT-26 and a visual task displaying paired high- and low-calorie food images alongside neutral controls. Oculomotor metrics included first fixation duration (FFD), fixation count (FC), and average fixation duration (AFD). Data were evaluated using EAT-26 threshold-based group stratification (lower-risk vs. risky eating behavior) and Spearman rank correlation analyses. **Results**: Global EAT-26 scores showed a significant correlation with a directional attentional bias index (rho = 0.29, *p* = 0.006). The Dieting subscale demonstrated no significant relationships with any oculomotor metrics. Conversely, Oral Control scores were significantly negatively correlated with the directional attentional bias index (rho = −0.27, *p* = 0.009) and positively tracked with low-calorie fixation counts (rho = 0.30, *p* = 0.004). Bulimia subscale scores showed a moderate positive correlation with the directional attentional bias index (rho = 0.26, *p* = 0.013), characterized by a significant decrease in low-calorie fixation counts (rho = −0.32, *p* = 0.002) and a strong increase in high-calorie fixation counts (rho = 0.36, *p* < 0.001) during sustained viewing. **Conclusions**: Subclinical eating pathology tendencies are associated with distinct implicit attentional profiles regarding food caloric density during sustained cognitive evaluation. Oral control drives visual prioritization of low-calorie stimuli to maintain inhibitory control, whereas bulimic tendencies reflect prolonged visual preoccupation with high-calorie reward cues. Eye-tracking represents a promising non-invasive research tool for exploring early cognitive correlates of eating-disorder risk.

## 1. Introduction

The limitations of current diagnostic and intervention strategies underscore the need for a shift toward identifying early cognitive markers. Contemporary theoretical frameworks increasingly conceptualize EDs not merely as behavioral disturbances, but as information-processing disorders [1,2,3,4,5,6,7,8]. This transdiagnostic perspective suggests that overvaluation of shape and weight drives selective attention to, and biased interpretation of, food-related stimuli [9]. Specifically, maladaptive eating behaviors are understood as consequences of fundamental cognitive–emotional processes—such as rigid, detail-focused processing (weak central coherence) and impaired set-shifting—that often operate outside of conscious awareness [10]. These neurocognitive features, including cognitive rigidity and pronounced attentional bias toward food-related stimuli, function as transdiagnostic features spanning multiple ED subtypes [11].

At the core of this altered cognitive processing is the differential valuation of nutritional stimuli based on their caloric density and macronutrient composition. Caloric density is predominantly governed by water, dietary fiber, fat, and sugar content [12]. Low-calorie foods (e.g., raw vegetables, unprocessed fruits) are typically characterized by high water and dietary fiber volume with minimal lipid concentrations, promoting homeostatic mechanosensory gastric distension and sustained satiety signaling without overactivating hedonic circuits. Conversely, high-calorie foods are dense in refined carbohydrates, simple sugars, and saturated fats. Foods rich in simple sugars provoke rapid glycemic fluctuations and immediate hedonic feedback, whereas dietary fats confer high palatability through unique oral texture, somatosensory perception, and delayed post-ingestive reward signaling [12,13].

Crucially, modern nutritional neuroscience demonstrates that the combination of high fat and refined sugar—a profile ubiquitous in ultra-processed foods but rarely found in nature—acts supra-additively to amplify neural reward responses within mesolimbic dopaminergic pathways (e.g., ventral striatum and nucleus accumbens) beyond the caloric value of either nutrient alone [13]. This hyper-potent reward activation confers potent visual “incentive salience” to energy-dense cues, transforming them into salient behavioral triggers that compete directly with top-down executive and inhibitory control networks [14]. In individuals vulnerable to eating pathologies, this neurochemical salience can manifest as an exaggerated approach-avoidance conflict: high-fat, high-sugar cues trigger intense appetitive anticipation and craving, while simultaneously provoking compensatory anxiety, guilt, or deliberate dietary restraint [11].

A critical barrier to elucidating these mechanisms is the reliance on explicit self-report measures (e.g., EAT-26), which are frequently compromised by social desirability bias, poor introspective access, and the clinical denial or lack of insight characteristic of ED populations [15,16,17,18]. Implicit cognitive assessments, which measure automatic responses, offer a more objective alternative [19,20]. Among these, eye-tracking technology has emerged as a robust, non-invasive method for quantifying visual attention in real-time [21]. By analyzing metrics such as first fixation duration (initial orienting) and total dwell time (sustained attention), researchers can differentiate between early attentional capture and subsequent strategic avoidance [22,23].

Recent eye-tracking evidence suggests temporally dynamic attentional patterns that vary by diagnostic category and caloric density. Individuals with AN often exhibit initial vigilance followed by rapid avoidance of high-calorie food cues—a strategy hypothesized to regulate food-related anxiety [24,25,26]. Conversely, BN and related binge-eating pathologies are frequently associated with an approach-avoidance conflict, where heightened reward sensitivity triggers initial engagement with high-calorie stimuli, potentially followed by avoidance driven by guilt or fear [11,27,28,29]. However, empirical findings remain inconsistent, often failing to cleanly differentiate between clinical categories or explain how these biases emerge in the pre-clinical stage [11,22].

Current research emphasizes a dimensional view of eating pathology, suggesting that attentional biases observed in clinical populations exist on a continuum and can be detected in subclinical samples [30,31]. Exploring these “analogue” populations provides a unique window into the early cognitive markers that precede full-threshold EDs, facilitating targeted prevention [32,33].

The primary objective of this study was to examine whether subclinical eating pathology dimensions (dieting, oral control, and bulimic tendencies), assessed using the EAT-26, are systematically associated with distinct temporal patterns of implicit visual attention toward high- versus low-calorie food stimuli in an unselected university cohort. By linking self-reported cognitive concerns to real-time gaze behavior across both initial orienting and sustained viewing stages, we aimed to clarify how caloric density and nutritional salience guide implicit attention prior to clinical onset.

As secondary hypotheses, we evaluated specific subscale-level behavioral divergences:

**Hypothesis 1** **(H1).** *Global eating attitudes (EAT-26 total) will correlate with overall visual engagement with food stimuli across fixation metrics.*

**Hypothesis 2** **(H2).** *Individuals with higher scores on the Dieting subscale will exhibit a greater attentional bias toward low-calorie food stimuli.*

**Hypothesis 3** **(H3).** *Individuals with higher scores on the Oral Control subscale will exhibit a greater attentional bias toward low-calorie food stimuli.*

**Hypothesis 4** **(H4).** *Higher Bulimia subscale scores will correlate with increased attentional orienting and sustained engagement toward high-calorie food cues, reflecting heightened reward sensitivity.*

## 2. Materials and Methods

### 2.1. Study Design and Ethics

This study utilized a cross-sectional observational design with an experimental eye-tracking task, incorporating psychometric questionnaires and eye-tracking assessments. The research protocol was approved by the Institutional Review Board of the University of Debrecen (Ethical Permission Number: UD-IP-2025/72). The participants were international students studying at the University of Debrecen, Hungary, recruited within a primary care setting (general practitioner clinic). Potential participants were informed through personal contact about the opportunity to voluntarily participate in the research either during their mandatory occupational health examination or while visiting the clinic for medical consultations. This recruitment strategy yielded an average, volunteering student population that effectively reflects the natural distribution and proportions of subclinical and clinical populations within this demographic. All participants provided informed consent prior to enrollment, and the study was conducted in accordance with the Declaration of Helsinki. Importantly, institutional framework conditions support this population; upon enrollment, all university students receive standardized information regarding available low-intensity psychological intervention services, which offer a well-defined institutional pathway to specialized clinical care if required.

### 2.2. Eligibility and Exclusion Criteria

To be eligible for inclusion in the study, participants were required to be active students at the university and eighteen years of age or older at the time of data collection.

Following enrollment, individuals were excluded from the final analysis if they failed to provide informed consent, failed to complete the eye-tracking assessment, or returned extensively incomplete psychometric questionnaires, such as datasets missing critical items on the Eating Attitudes Test-26 (EAT-26) or foundational demographic forms.

### 2.3. Psychometric Assessment and Group Classification

After a short demographic questionnaire, participants were asked to complete the Eating Attitudes Test-26 (EAT-26) to assess eating disorder symptomatology. The EAT-26 is a widely used standardized self-report instrument that measures eating-related attitudes and behaviors across three subscales: Dieting, Oral Control, and Bulimia. The Dieting subscale reflects concerns about dieting, preoccupation with being thinner, and avoidance of high-calorie foods. The Oral Control subscale reflects perceived self-control over eating and perceived pressure from others regarding eating, while the Bulimia subscale captures binge-related concerns and food preoccupation, including thoughts and behaviors linked to loss of control over eating [34].

Based on standardized EAT-26 scoring, participants were stratified into two groups: a risky eating behavior group and a lower-risk (lower EAT-26) group. The risky group was operationally defined by an EAT-26 score ≥ 20, consistent with established screening criteria indicating elevated risk for eating-disorder symptomatology. The control group was defined by an EAT-26 score < 20. Elevated scores were further characterized according to the Dieting, Oral Control, and Bulimia/Food Preoccupation subscales of the EAT-26. This classification approach is consistent with prior validation and subtyping research using the EAT-26 [35].

### 2.4. Eye-Tracking Apparatus and Stimuli

Eye-movement data were acquired via a desktop-mounted, video-based infrared eye tracker (Tobii Pro Fusion, Tobii AB, Stockholm, Sweden) utilizing a 120 Hz sampling frequency. Stimuli were presented on a 15.6-inch display with a native resolution of 1920 × 1080 pixels (Full HD). To ensure optimal tracking accuracy and data integrity, participants were positioned at a viewing distance of approximately 60 cm.

Visual stimuli consisted of 20 digital images selected from the Food-pics Extended database, a standardized repository featuring normative ratings for valence, arousal, palatability, and desire to eat [36]. The experimental stimuli were arranged into 10 pairs and divided into two distinct categories. The experimental stimuli consisted of five food-image pairs designed to compare caloric density; in each instance, a high-calorie item (e.g., pizza) was presented on the right side of the screen, while a low-calorie item (e.g., salad) was presented on the left. The control stimuli consisted of five pairs of neutral objects, such as office supplies, to establish a baseline for gaze behavior independent of nutritional content.

### 2.5. Experimental Procedure

Upon arrival at the laboratory, participants provided informed consent and completed the psychometric assessments. The eye-tracking session took place in a quiet, distraction-free room. Prior to the task, a standard calibration procedure was performed; only participants yielding > 70% valid calibration data were retained for analysis. Participants were instructed to view the screen naturally, as if they were browsing pictures on a computer monitor. The eye-tracking task consisted of 10 continuous trials, with each stimulus pair presented for 5000 ms. Stimulus pairs were presented in a fixed block order, with all five food stimulus pairs presented first, followed by the five neutral object pairs.

### 2.6. Data Extraction and Processing

Eye-tracking data were preprocessed and extracted utilizing Tobii Pro Lab software (version 25.23). Predefined Areas of Interest (AOIs) were digitally mapped around the key objects within each image. While multiple oculomotor metrics were computed, attentional processing was primarily operationalized using average fixation duration. This metric was selected to reflect overall, sustained attentional engagement with the stimuli rather than distinct temporal stages of visual processing. For each participant, the average fixation durations were calculated separately within the defined AOIs for high-calorie, low-calorie, and neutral stimuli.

Attentional bias toward food stimuli was operationalized using a difference score, which was calculated by subtracting the average fixation duration for low-calorie items from the average fixation duration for high-calorie items. Additionally, absolute bias scores were computed to capture the overall magnitude of attentional engagement, regardless of whether the bias was directed toward high- or low-calorie stimuli.

### 2.7. Statistical Analysis

Descriptive statistics were computed for the final valid sample of 89 participants after data screening, and the internal consistency of the EAT-26 was evaluated, demonstrating excellent reliability within the current sample (Cronbach’s alpha = 0.90). Prior to conducting inferential analyses to compare EAT-26 scores between the control and risky groups, preliminary assumption checks were performed. Data distribution normality was assessed using the Shapiro–Wilk (W = 0.92, *p* < 0.01), Kolmogorov–Smirnov (*p* = 0.01), and Anderson–Darling (*p* < 0.01) tests, which collectively indicated that the data significantly deviated from normality [37,38,39]. Furthermore, Levene’s test indicated unequal variances between the groups (*p* < 0.05), violating the assumption of homogeneity of variance [40].

Because the assumptions for standard parametric testing were violated regarding both normality and homogeneity of variance, both Welch’s *t*-test and the non-parametric Mann–Whitney U test were deemed appropriate and subsequently employed to compare EAT-26 scores between the risky and control groups [41,42]. To examine systematic lateralization bias, paired-samples *t*-tests were conducted for neutral stimuli by comparing left and right presentations across average fixation duration, first fixation duration, and fixation count. For hypothesis testing, Spearman rank correlation analyses were used to examine the relationships between EAT-26 total scores, subscale scores, and attentional bias indices derived from average fixation duration [43]. Finally, rank correlations were calculated for the overall attentional bias score, specific food-category measures, and fixation-count indices.

All statistical analyses were performed using the Jamovi statistical software (version 2.6.45; Sydney, Australia) with significance defined at *p* < 0.05 [44].

## 3. Results

### 3.1. Participant Characteristics and Psychometric Validation

**Figure 1:**
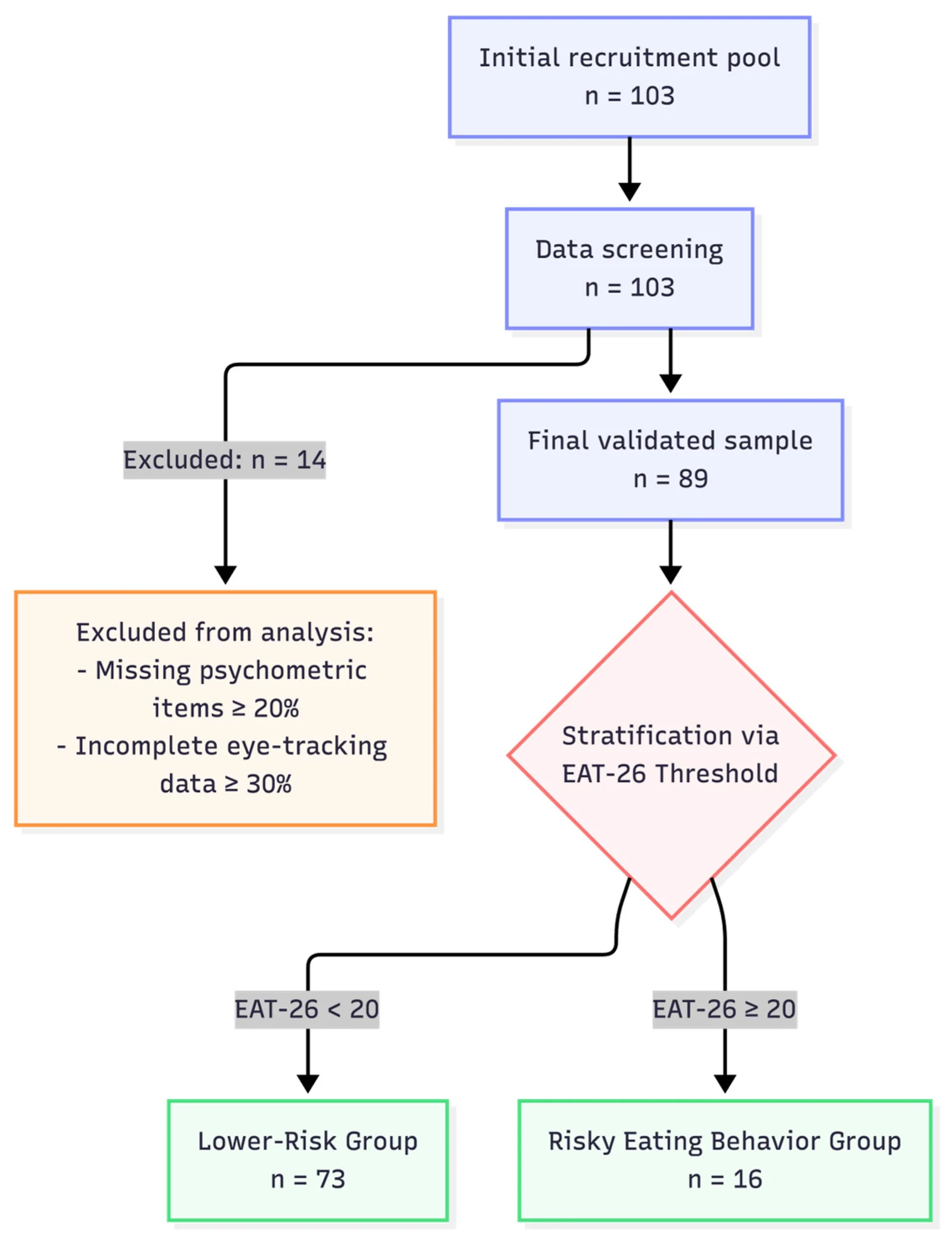
Participant Selection and Enrollment Flowchart. The diagram details the initial recruitment pool (n = 103), the specific reasons for exclusion (missing psychometric data ≥ 20% and incomplete eye-tracking calibration ≥ 30%), and the final validated sample size (n = 89) utilized for statistical analysis.

To illustrate the study’s selection process, a PRISMA-style participant flow diagram is presented in Figure 1, detailing enrollment, exclusions, and final allocations. From the initial pool of 103 recruited individuals the final validated sample comprised 89 participants following data screening, which excluded 14 individuals with missing psychometric items (≥20%) or incomplete eye-tracking data (≥30%).

**Figure 2:**
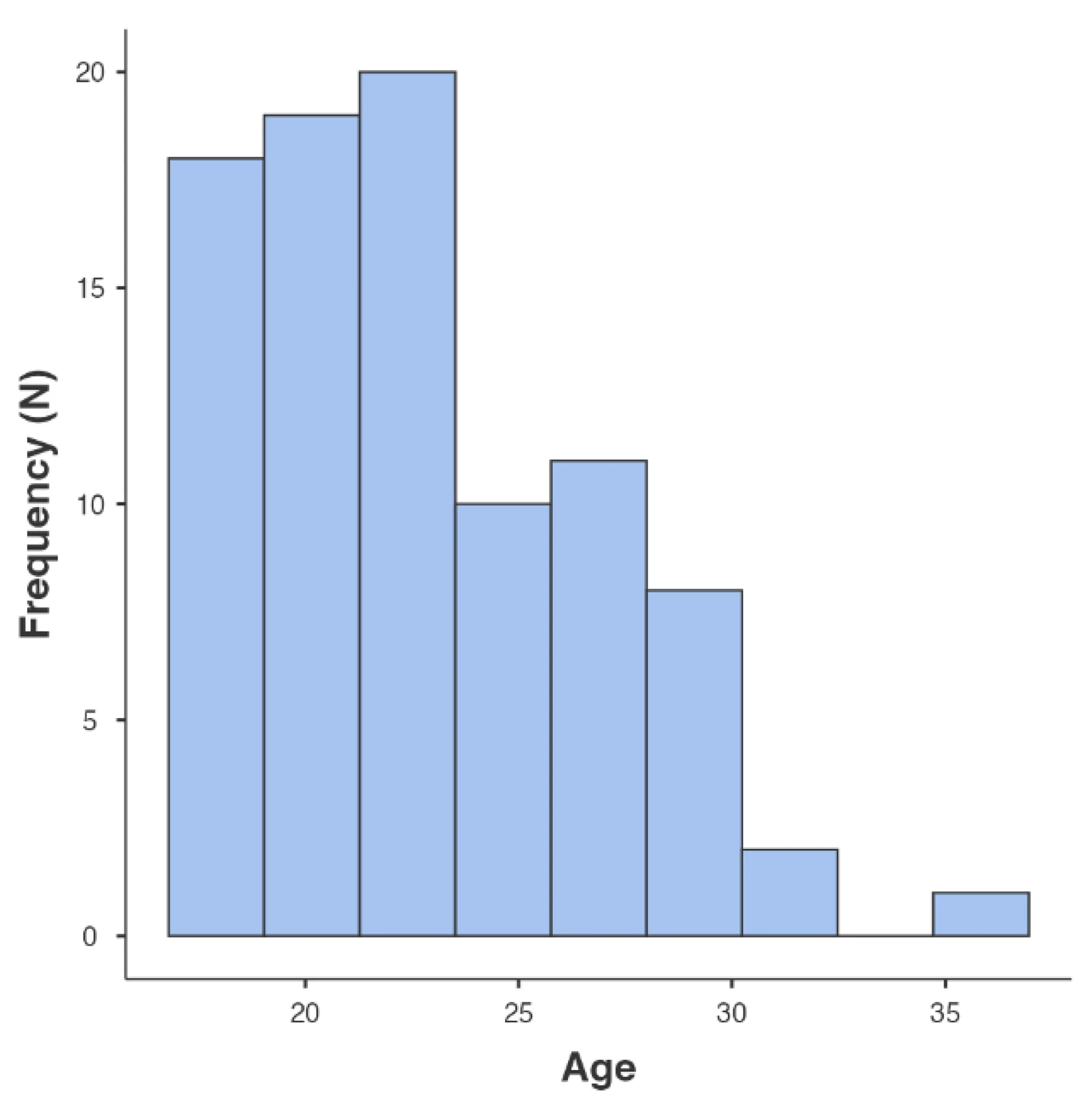
Histogram of Age Distribution Across the Validated Sample. The x-axis represents participant age in years, and the y-axis indicates the absolute frequency of participants. The sample demonstrated a right-skewed distribution, primarily comprising young university adults (Mean = 22.88 years, SD = 3.80, Range = 18–35).

The cohort exhibited a mean age of 22.88 years (SD = 3.80, Median = 22, see Figure 2). Gender distribution consisted of 48 females, 40 males, and 1 missing response. Based on the standardized Eating Attitudes Test-26 (EAT-26) clinical threshold, participants were stratified into a lower-risk group (n = 73, EAT-26 < 20) and a risky eating behavior group (n = 16, EAT-26 ≥ 20).

Descriptive data revealed distinct distributional characteristics between the groups. The lower-risk group exhibited low overall scores (M = 6.23, SD = 5.71, Median = 4, Range = 0–19) with a significant positive skewness of 0.82, as confirmed by a significant Shapiro–Wilk test (W = 0.88, *p* < 0.01). Conversely, the risky eating behavior group demonstrated elevated scores (M = 30.44, SD = 9.41, Median = 27, Range = 20–52) and did not significantly deviate from a normal distribution (W = 0.91, *p* = 0.116), despite a mild positive skewness of 0.86.

Robust inferential analyses were conducted to evaluate group differences. Both Welch’s *t*-test (t(17.49) = −9.90, *p* < 0.01) and the non-parametric Mann–Whitney U test (U = 0, *p* < 0.01) demonstrated that the risky behavior group had significantly higher EAT-26 scores than the lower-risk group.

**Figure 3:**
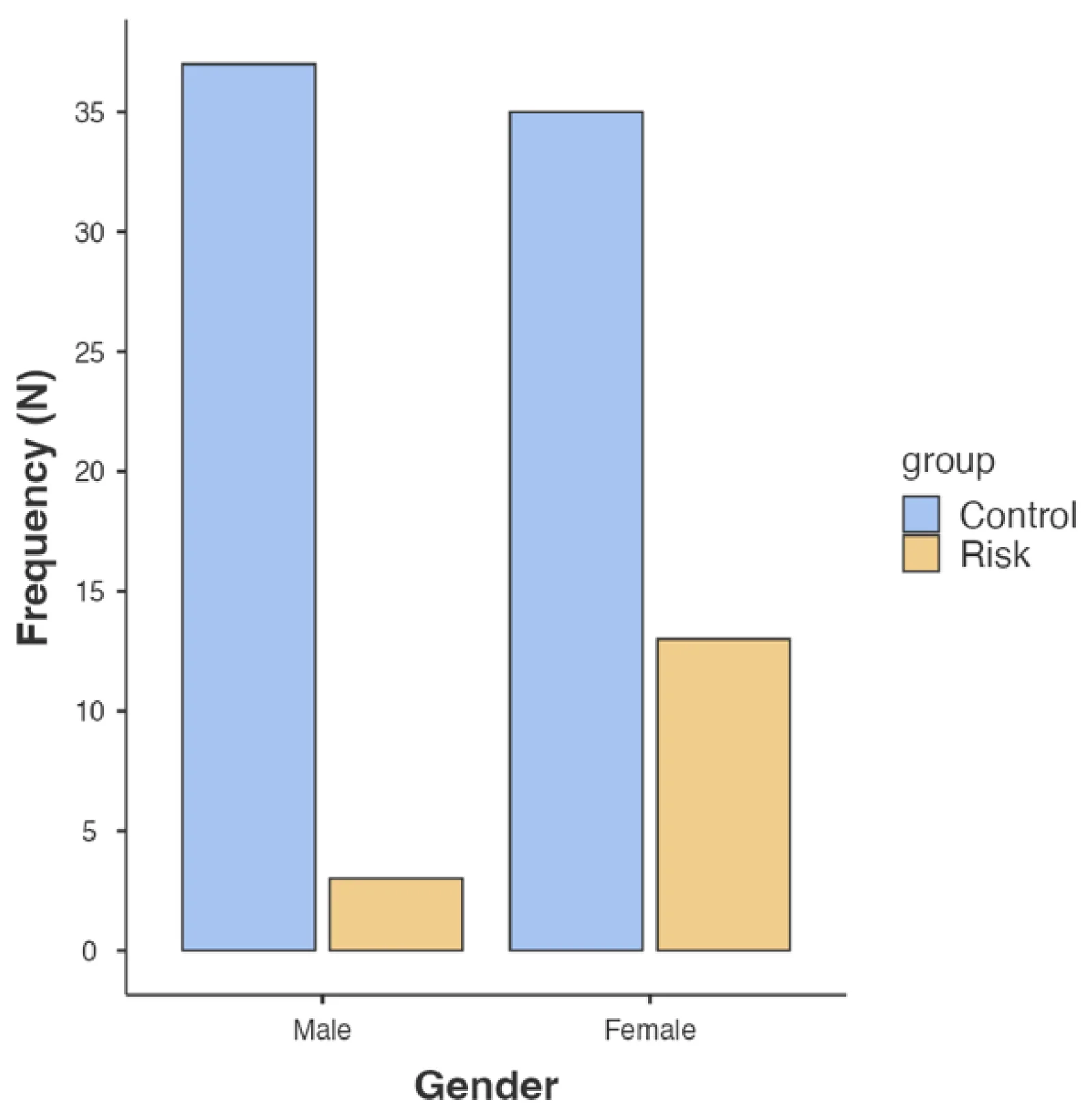
Grouped Bar Chart of Gender Distribution Stratified by EAT-26 Experimental Group. The chart compares the absolute counts of male and female participants across the Lower-Risk (EAT-26 < 20) and Risky Eating Behavior (EAT-26 ≥ 20) groups. Females were significantly overrepresented within the risky group (*p* = 0.009), consistent with epidemiological trends in subclinical eating pathology.

Demographic comparisons between the groups via Mann–Whitney U tests revealed no significant differences regarding age (*p* = 0.429). However, a statistically significant association emerged between group membership and gender (*p* = 0.009, effect size = 0.326), indicating that female participants were significantly overrepresented within the risky eating behavior group (see Figure 3).

Anthropometric and self-reported dietary characteristics were evaluated across both groups. The lower-risk group (Mean age = 22.74, SD = 3.81) exhibited a mean height of 170.83 cm (SD = 9.74) and a mean weight of 72.33 kg (SD = 16.86), yielding an average Body Mass Index (BMI) of 24.64 kg/m^2^ (SD = 4.59). The risky eating behavior group (Mean age = 23.50, SD = 3.85) presented a mean height of 170.07 cm (SD = 6.57) and a mean weight of 68.29 kg (SD = 15.47), corresponding to an average BMI of 23.81 kg/m^2^ (SD = 4.96). Mann–Whitney U tests revealed no significant differences between groups regarding BMI (U = 447.00, *p* = 0.749), indicating baseline anthropometric equivalence.

Regarding general dietary characteristics, the lower-risk group reported moderate satisfaction with their overall eating behaviors (M = 3.40, SD = 0.89 on a 5-point scale) and body shape (M = 2.78, SD = 1.00). Conversely, the risky eating behavior group reported slightly lower satisfaction with their eating behaviors (M = 3.00, SD = 1.15) and similar ratings for body shape satisfaction (M = 2.88, SD = 1.36). Mann–Whitney U comparisons indicated no significant difference in eating behavior satisfaction (U = 448.50, *p* = 0.937) and no significant difference in body shape satisfaction (U = 583.00, *p* = 0.507).

### 3.2. Experimental Stimuli and Spatial Neutrality Validation

To ensure that subsequent oculomotor assessments reflected semantic processing of nutritional content rather than general visual asymmetries or spatial lateralization biases, paired-samples *t*-tests were conducted on the neutral stimulus pairs. Visual attention allocation did not differ significantly between the left- and right-presented neutral objects across any extracted metric. Specifically, average fixation duration was identical for left (M = 2.30 s, SD = 0.67) and right (M = 2.30 s, SD = 0.58) neutral stimuli (t(88) = −0.19, *p* = 0.85). Fixation counts also showed no significant differences between left (M = 7.76, SD = 2.16) and right (M = 7.92, SD = 2.41) presentations (t(88) = −0.71, *p* = 0.48). Finally, initial orienting, measured by first fixation duration, was statistically equivalent between left (M = 0.25 s, SD = 0.22) and right (M = 0.23 s, SD = 0.10) neutral stimuli (t(88) = 0.80, *p* = 0.423). These findings confirm the absence of confounding spatial lateralization biases.

### 3.3. Oculomotor Profiles Across Experimental Conditions

Descriptive patterns for the primary eye-tracking metrics—first fixation duration (FFD), fixation count (FC), and average fixation duration (AFD)—revealed divergent trends based on caloric density and group status. For low-calorie food stimuli, the risky eating behavior group demonstrated prolonged initial orienting (FFD: M = 0.30 s, SD = 0.29), more frequent visual engagement (FC: M = 10.34, SD = 3.70), and longer overall viewing times (AFD: M = 2.84 s, SD = 0.88) relative to the lower-risk group (FFD: M = 0.23 s, SD = 0.06; FC: M = 8.87, SD = 3.18; AFD: M = 2.50 s, SD = 0.91).

Conversely, when exposed to high-calorie food stimuli, the lower-risk group displayed higher sustained engagement than the risky group, characterized by elevated fixation counts (Controls: M = 8.24, SD = 3.14; Risky: M = 7.56, SD = 3.36) and longer average viewing times (Controls: M = 2.25 s, SD = 0.91; Risky: M = 2.10 s, SD = 0.90). Initial orienting to high-calorie cues remained marginally higher in the risky group (M = 0.28 s, SD = 0.16) compared to controls (M = 0.25 s, SD = 0.11). Group-specific descriptive eye-tracking metrics are summarized in Table 1.

### 3.4. Hypothesis Testing and Inferential Oculomotor Correlations

Attentional bias was operationalized via a directional difference score, calculated by subtracting the average fixation duration of low-calorie items from that of high-calorie items. Positive scores indicated a relative preference for high-calorie images, whereas negative scores indicated a preference for low-calorie images. Absolute bias scores were also calculated to capture the total magnitude of attentional deviation. Spearman rank correlation analyses were executed to test the four primary hypotheses regarding the relationship between explicit psychometric dimensions and implicit visual deployment.

#### 3.4.1. Global Eating Attitudes (H1)

Spearman correlation analysis provided borderline clear support for Hypothesis 1, revealing a statistically significant positive relationship between global EAT-26 total scores and the directional attentional bias index (rho = 0.29, *p* = 0.006). This confirms that higher EAT-26 scores are associated with greater attentional bias between low- and high-calorie foods.

#### 3.4.2. Dieting Subscale (H2)

Hypothesis 2 was not supported. Dieting subscale scores demonstrated no significant correlation with the primary attentional bias index (rho = 0.09, *p* = 0.429). Regarding early visual orienting, Dieting subscale scores demonstrated no significant relationship with first fixation duration (FFD) for either low-calorie (rho = −0.02, *p* = 0.982) or high-calorie (rho = 0.04, *p* = 0.704) stimuli. Similarly, no statistically significant associations emerged for fixation counts across low-calorie (rho = −0.06, *p* = 0.550) or high-calorie (rho = 0.14, *p* = 0.185) conditions, indicating that dieting tendencies did not reliably track with any eye-tracking metrics.

#### 3.4.3. Oral Control Subscale (H3)

Hypothesis 3 was supported. A significant negative correlation emerged between Oral Control scores and the primary attentional bias index (rho = −0.27, *p* = 0.009), showing that individuals with higher oral control exhibit greater relative sustained attention toward low-calorie food stimuli. Stage-specific analyses indicated that this bias was driven by sustained engagement rather than initial orienting. Oral Control scores were not correlated with FFD for low-calorie (rho = −0.10, *p* = 0.331) or high-calorie (rho = −0.04, *p* = 0.705) stimuli, nor with high-calorie fixation counts (rho = −0.17, *p* = 0.108). However, a significant positive correlation was observed between Oral Control scores and low-calorie fixation counts (rho = 0.30, *p* = 0.004) (see Table 2).

#### 3.4.4. Bulimia Subscale (H4)

Hypothesis 4 was partially supported. Bulimia subscale scores exhibited a significant, moderate positive correlation with the directional attentional bias index (rho = 0.26, *p* = 0.013), linking higher bulimic tendencies to a sustained visual preference for high-calorie cues. In terms of temporal dynamics, no significant correlations were detected between Bulimia scores and initial orienting (FFD) for either low-calorie (rho = 0.18, *p* = 0.089) or high-calorie (rho = 0.06, *p* = 0.571) stimuli. Instead, the effect was driven by sustained engagement metrics: Bulimia scores were significantly negatively correlated with low-calorie fixation counts (rho = −0.32, *p* = 0.002) and strongly positively correlated with high-calorie fixation counts (rho = 0.36, *p* < 0.001).

#### 3.4.5. Qualitative Gaze Topography and Heatmap Analysis

**Figure 4:**
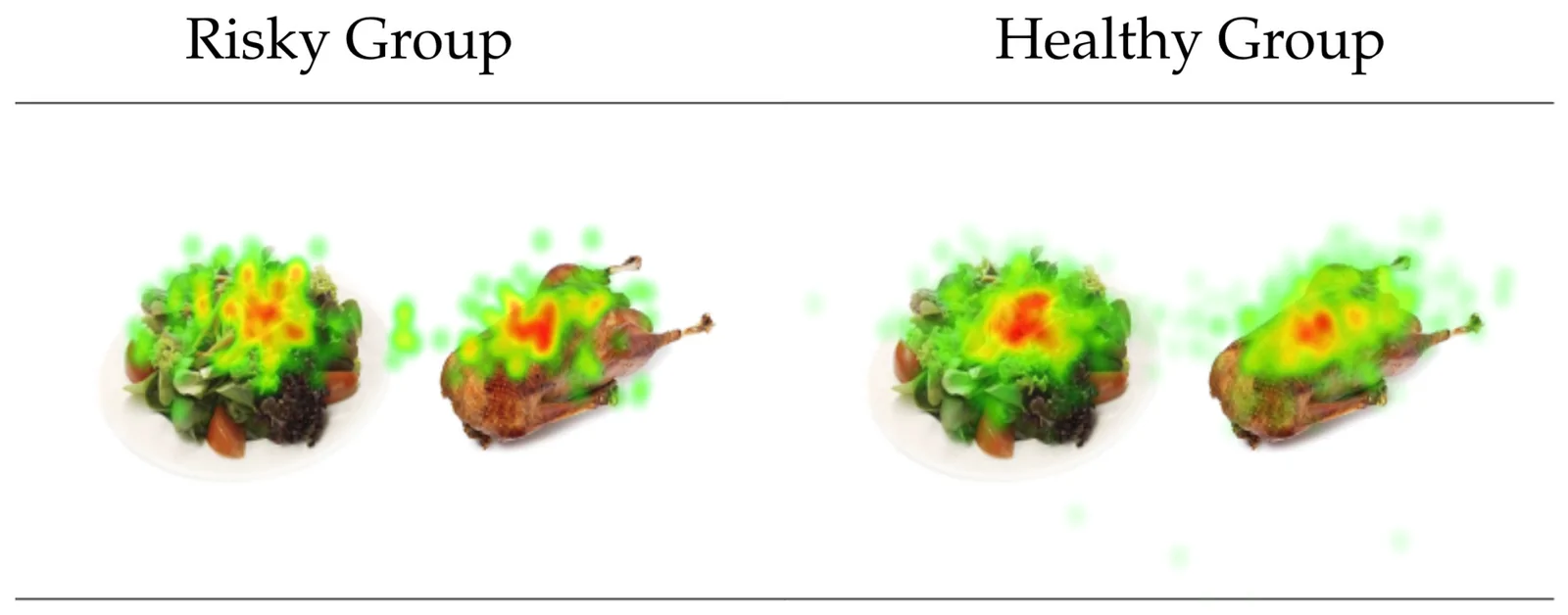
Qualitative Eye-Tracking Heatmaps of Visual Attention Distribution (Salad vs. Roast Chicken). The heatmaps visualize aggregated gaze duration and fixation density (warmer colors denote higher visual engagement) for a representative Lower-Risk participant (**right**) and a Risky Eating Behavior participant (**left**). The lower-risk participant exhibits higher gaze density on the low-calorie stimulus (salad), while the risky participant displays pronounced fixation clustering on the high-calorie stimulus (roast chicken).

**Figure 5:**
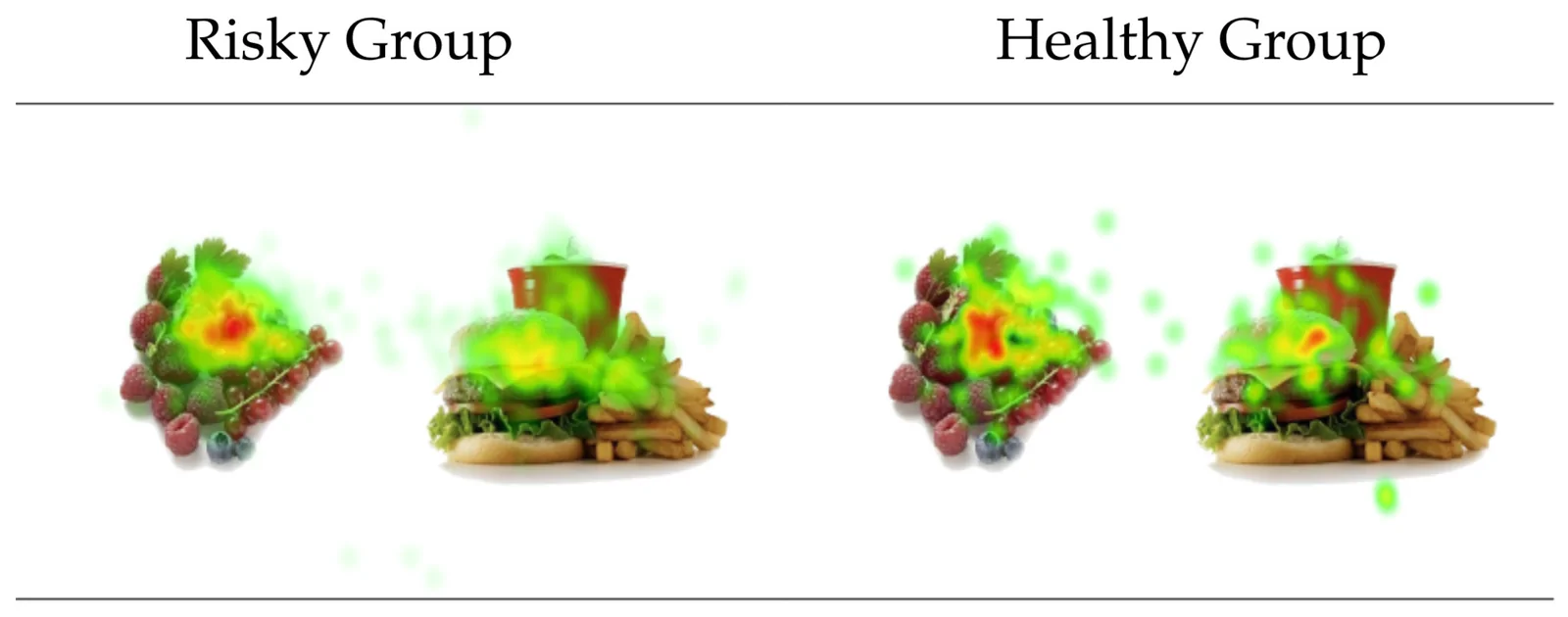
Qualitative Eye-Tracking Heatmaps of Visual Attention Distribution (Fruit vs. Fast Food). Warmer colors indicate greater cumulative visual dwell time. Consistent with quantitative subscale findings, the representative Risky Eating Behavior participant demonstrates sustained visual preoccupation targeted at the high-calorie fast food cues compared to the lower-risk control.

To complement the group-level statistical findings, qualitative evaluation of individual gaze fixation heatmaps was conducted to provide illustrative case examples from the respective groups (see Figure 4 and Figure 5). For the selected participant from the Lower-Risk (“Healthy”) Group visual fixations show a high density clustered around the low-calorie food regions, such as salads and fruits. Conversely, the heatmap from the selected participant within the Risky Eating Behavior Group displays a concentrated gaze density on the high-calorie food regions, including the roast chicken and fast food items. While these individual topographies provide a visual demonstration of divergent explicit-implicit profiles, they represent single-subject examples and should be interpreted in conjunction with the broader aggregate metrics detailed in Table 1, which capture the full variance across the entire sample.

## 4. Discussion

### 4.1. Interpretation of Distributional and Demographic Configurations

The psychometric findings demonstrate a clear delineation between the lower-risk and the risky eating behavior groups. The control group’s compressed distribution toward lower values and positive skewness indicate a floor effect. This pattern is characteristic of non-clinical populations where explicit eating concerns are minimal. Conversely, the risky group’s higher mean and median reflect a systematic shift in central tendency rather than an artifact driven by isolated outliers.

Demographically, the lack of age differences indicates that the current findings are independent of age-related variations within this university cohort. However, the significant overrepresentation of females within the risky eating behavior group (*p* = 0.009, effect size = 0.326) aligns with international epidemiological data indicating that women exhibit a heightened risk profile for eating pathology [45].

### 4.2. Methodological Rigor and Visual Neutrality

The paired-samples *t*-tests on neutral stimuli demonstrated no significant differences in fixation counts, average fixation durations, or initial orienting between left and right presentations. This absolute spatial symmetry is a critical methodological validation, confirming that participants did not possess a systematic spatial or lateralization bias. Consequently, the divergent eye-tracking behaviors observed during food presentations can be confidently attributed to the semantic and motivational salience of the nutritional cues, rather than mechanical artifacts or spatial confounds.

### 4.3. Explicit-Implicit Divergence and Subscale Attentional Typologies

The significant association observed for Hypothesis 1 (rho = 0.29, *p* = 0.006) is consistent with transdiagnostic information-processing models, which posit that cognitive overvaluation of shape, weight, and eating drives selective attention toward disorder-relevant stimuli [8,9]. Individuals with higher subclinical eating concerns assign greater motivational salience to food cues, which alters their visual allocation. This finding is consistent with cognitive models mentioned previously, which propose that maladaptive eating behaviors are maintained by biased information processing, including selective attention toward disorder-relevant stimuli; this has been directly substantiated by eye-tracking paradigms capturing enhanced fixation maintenance on salient food items [28].

From a dual-process framework, these results indicate that implicit cognitive processes partially mirror underlying explicit attitudes [46]. However, the modest magnitude of this correlation emphasizes that the explicit and implicit systems do not completely overlap. This finding aligns with dual-process models stating that explicit questionnaires and implicit gaze metrics capture distinct facets of cognition, particularly in domains susceptible to social desirability and limited introspective access [47].

The failure of the Dieting subscale (H2) to correlate with any eye-tracking metrics suggests an “intention-implementation gap.” Cognitive dietary restraint represents a reflective, explicit goal that may not automatically translate into bottom-up, implicit attentional prioritization. These subtle cognitive processes may require clinical-threshold pathology or highly restrictive conditions to influence automatic oculomotor behaviors.

In contrast, the Oral Control subscale (H3) was significantly associated with a negative attentional bias (rho = −0.27, *p* = 0.009) and a higher low-calorie fixation count (rho = 0.30, *p* = 0.004). This asymmetry shows that oral control reflects a specific attentional prioritization of “safe” or low-calorie food stimuli rather than a general avoidance of high-calorie foods. This pattern may capture top-down compensatory regulatory mechanisms, where individuals actively direct visual attention toward diet-congruent cues to maintain inhibitory control over eating.

Finally, the Bulimia subscale (H4) demonstrated a moderate positive correlation with high-calorie attentional bias (rho = 0.26, *p* = 0.013), characterized by a reduction in low-calorie fixations (rho = −0.32, *p* = 0.002) and an increase in high-calorie fixations (rho = 0.36, *p* < 0.001). This pattern is consistent with theoretical models of bulimic symptomatology, which propose heightened reward sensitivity toward high-calorie foods alongside reduced engagement with diet congruent or “safe” food cues, a trend strongly tied to food-specific reward sensitivity and disinhibited eating phenotypes [48]. Crucially, the absence of an effect on first fixation duration indicates that these biases may not primarily operate during automatic early orienting stages but rather emerge during later stages of sustained visual engagement. This interpretation is consistent with free-viewing eye-tracking evidence linking dwell-time-based attentional bias to high-calorie foods with eating-disorder symptoms, and with models distinguishing bottom-up food-cue salience from top-down goal-directed attentional control [49,50].

When contextualizing these attentional biases, it is crucial to consider the underlying mechanisms of appetite and physiological satiety [51]. In healthy cohorts, orexigenic hormones (e.g., ghrelin) typically enhance visual attention to food cues during states of hunger, whereas anorexigenic signaling (e.g., leptin, gastric distension) actively suppresses this attentional bias once satiety is achieved [51]. However, individuals exhibiting higher subclinical eating pathology or susceptibility to weight gain frequently demonstrate an impaired ability to downregulate visual attention to high-calorie food cues even when physiologically satiated. This sustained visual engagement despite metabolic fullness is a primary cognitive mechanism linked to disinhibited eating, reward-driven caloric surplus, and subsequent weight gain [51].

Similarly, evaluating the unique profile of different energy sources is critical when discussing caloric density. For instance, although alcoholic beverages were not utilized as stimuli in the present study, alcohol represents a highly dense macronutrient energy source (yielding approximately 7 kcal/g) that warrants theoretical consideration [52]. Unlike solid foods, liquid calories like alcohol fail to trigger standard mechanical and hormonal satiety responses (e.g., gastric distension). Because alcohol bypasses these early satiation mechanisms, it fails to meaningfully reduce the hedonic appeal or visual salience of subsequent food cues, which heavily contributes to a positive energy balance and weight gain in broader dietary contexts [52].

From a practical and public health perspective, these findings hold valuable implications for early intervention within university frameworks. Because the institutional ecosystem under study already provides all incoming students with standardized information on low-intensity psychological intervention services, a clear infrastructure for early mental health support is already in place. Objective, non-invasive cognitive markers—such as the eye-tracking metrics explored here—represent a promising, exploratory avenue that could, pending replication in larger and more diverse samples, eventually complement these existing low-threshold frameworks. At this stage, eye-tracking should be regarded as a research tool rather than a validated screening instrument; further work is needed before any such application to early identification pathways could be considered.

### 4.4. Clinical and Pharmacological Implications

From a translational perspective, the distinct oculomotor profiles identified in this study hold significant implications for evaluating both dietary and pharmacological interventions. Recent neuroimaging and clinical research indicate that pharmacological treatments for obesity and disordered eating, particularly Glucagon-Like Peptide-1 (GLP-1) receptor agonists (e.g., liraglutide, semaglutide), act directly on mesolimbic reward centers to reduce the visual and neural incentive salience of high-calorie foods [53]. Consequently, eye-tracking metrics could serve as a valuable, non-invasive biomarker to monitor the neurocognitive efficacy of GLP-1 therapies in reducing cue-induced cravings [53].

Furthermore, these findings are relevant to nutritional interventions, such as high-protein (low-calorie) diets. High dietary protein enhances endogenous satiety signaling (including natural GLP-1 and PYY secretion), which mimics pharmacological effects by dampening the top-down visual attentional pull toward highly palatable, energy-dense foods [53]. By mapping these implicit visual behaviors in a subclinical cohort, our findings suggest that tracking oculomotor changes could provide objective, real-time insights into how effectively specialized diets or GLP-1 analogs successfully suppress the cognitive preoccupation with high-calorie cues prior to overt behavioral weight loss.

### 4.5. Limitations and Future Directions

Several limitations must be acknowledged when interpreting these findings. First, because this study evaluated a subclinical sample, the number of individuals exhibiting severe, clinical-threshold symptomatology (the risky eating behavior group, n = 16) was relatively small. This limits the generalizability of these findings to formal diagnostic cohorts (e.g., Anorexia Nervosa or Bulimia Nervosa). Consequently, the present study should be viewed as a foundational, exploratory step intended to guide future research toward incorporating larger sample sizes and dedicated clinical populations.

Second, a notable methodological limitation concerns the fixed presentation format. High-calorie stimuli were always presented on the right side of the screen and low-calorie stimuli always on the left, and all food stimulus pairs were presented in a fixed block preceding the neutral pairs, without counterbalancing or randomization of stimulus side or block order. Although the paired-samples comparisons of neutral stimuli did not detect a left-right spatial asymmetry, a fixed pairing of caloric density with screen side and a fixed block order cannot fully rule out order or lateralization effects, and future studies should counter-balance stimulus side and block order.

Third, state-dependent motivational factors were not fully controlled; several participants completed the eye-tracking assessment during lunchtime and reported experiencing hunger. Because physiological hunger profoundly alters visual incentive salience, this lack of strict metabolic control represents a significant confounding factor that may have influenced the magnitude of the observed attentional biases.

Fourth, because gender was significantly associated with risk-group membership (*p* = 0.009), the observed associations between EAT-26 scores and attentional bias indices may be partly confounded by gender. While this female overrepresentation is in line with epidemiological data, sensitivity analyses controlling for gender, and, where available, age and self-reported hunger state, are recommended before the correlational findings are interpreted as reflecting purely eating-pathology-specific attentional processes.

Fifth, the explicit measures relied on self-report, which is inherently vulnerable to social desirability, acquiescence bias, and extreme response tendencies. Sixth, a preference for low-calorie stimuli among certain participants may stem from healthy lifestyle practices, such as fitness training or specialized diets (e.g., oil-free or sugar-free), rather than subclinical eating pathology. Seventh, the controlled laboratory setting limits the ecological validity of the findings compared to real-world environments. Eighth, while eye-tracking is widely used, it remains debated whether overt gaze parameters fully and directly capture underlying covert cognitive states and motivational processes, as these metrics are sensitive to task contexts and perceptual constraints.

Methodologically, this study relied primarily on average fixation duration to operationalize sustained attention, which may overlook subtle temporal shifts in visual processing. Furthermore, utilizing the standard EAT-26 cut-off score imposed a categorical split on a continuous, dimensional construct, which may have reduced statistical sensitivity to gradient-like relationships across the continuum of eating pathology. In addition, given that multiple subscale-by-metric correlations were examined, the associations reported here should be considered exploratory, and the possibility of findings arising from multiple testing cannot be excluded.

Finally, it is extremely important to clarify that while these findings present promising cognitive correlates of eating-disorder risk, eye-tracking technology must currently be considered a research tool, not a validated instrument for screening in clinical practice. Extensive methodological refinement is required before any application to early identification or clinical intervention pathways could be considered.

## 5. Conclusions

This study suggests that subclinical eating pathology tendencies are associated with distinct, implicit attentional profiles regarding food caloric density. By integrating the EAT-26 psychometric instrument with objective eye-tracking metrics, these findings are consistent with transdiagnostic information-processing models within a subclinical, non-clinical population. The significant correlation between global eating attitudes and the directional attentional bias index confirms that elevated eating concerns alter visual information processing and automatic motivational salience toward food stimuli.

Crucially, the findings reveal distinct behavioral divergences across specific psychometric subscales, manifesting primarily during sustained evaluation stages rather than early, automatic visual orienting. Elevated oral control scores drive a targeted visual prioritization of low-calorie stimuli, which reflects top-down compensatory mechanisms aimed at maintaining inhibitory control over “safe” food cues. Conversely, higher bulimic tendencies correlate with an increased attentional bias and higher fixation counts toward high-calorie stimuli, demonstrating prolonged visual preoccupation and heightened reward sensitivity. The total absence of oculomotor correlations with the dieting subscale underscores an explicit-implicit “intention-implementation gap” characteristic of subclinical cohorts.

In conclusion, this research highlights the potential utility of eye-tracking technology as a promising, non-invasive research tool for exploring early cognitive correlates that may precede full-threshold eating disorders, warranting further investigation for targeted prevention. To resolve the specific boundaries of this study—namely the reliance on a subclinical university cohort, potential state-dependent hunger confounds, and the limitations of categorical stratification—future research must employ continuous modeling within larger, clinically diverse samples.

## Figures and Tables

**Table 1:** Descriptive Oculomotor Metrics across Group and Stimulus Typologies.

Metric and Stimulus Type	Lower-Risk Group (n = 73)	Risky Eating Behavior (n = 16)
**First Fixation Duration (s)**		
Low-Calorie Food	0.23 (SD = 0.06)	0.30 (SD = 0.29)
High-Calorie Food	0.25 (SD = 0.11)	0.28 (SD = 0.16)
Neutral Stimuli (Left)	0.25 (SD = 0.22)	0.25 (SD = 0.11)
Neutral Stimuli (Right)	0.23 (SD = 0.10)	0.26 (SD = 0.09)
**Fixation Count (n)**		
Low-Calorie Food	8.87 (SD = 3.18)	10.34 (SD = 3.70)
High-Calorie Food	8.24 (SD = 3.14)	7.56 (SD = 3.36)
Neutral Stimuli (Left)	7.76 (SD = 2.16)	7.78 (SD = 1.75)
Neutral Stimuli (Right)	7.92 (SD = 2.41)	8.20 (SD = 1.58)
**Average Fixation Duration (s)**		
Low-Calorie Food	2.50 (SD = 0.91)	2.84 (SD = 0.88)
High-Calorie Food	2.25 (SD = 0.91)	2.10 (SD = 0.90)
Neutral Stimuli (Left)	2.30 (SD = 0.67)	2.22 (SD = 0.47)
Neutral Stimuli (Right)	2.30 (SD = 0.58)	2.33 (SD = 0.50)

**Table 2:** Correlation Matrix of the Relationships Between Oral Control Scores and Eye-Tracking Metrics (FFD, First Fixation Duration; and FC, Fixation Count) for Low- and High-Calorie Stimuli.

		Oral Control	Low FFD	High FFD	Low FC	High FC
**Oral Control**	Spearman’s rho	—				
	*df*	—				
	*p*-value	—				
**Low FFD**	Spearman’s rho	−0.1	—			
	*df*	87	—			
	*p*-value	0.331	—			
**High FFD**	Spearman’s rho	−0.04	0.23	—		
	*df*	87	87	—		
	*p*-value	0.705	0.032	—		
**Low FC**	Spearman’s rho	0.3	−0.34	−0.15	—	
	*df*	87	87	87	—	
	*p*-value	0.004	<0.001	0.157	—	
**High FC**	Spearman’s rho	−0.17	0.09	−0.18	−0.43	—
	*df*	87	87	87	87	—
	*p*-value	0.108	0.385	0.084	<0.001	—

## Data Availability

The data presented in this study are available on request from the corresponding author. The data are not publicly available due to privacy restrictions.

## Abbreviations

The following abbreviations are used in this manuscript:

AFD	Average fixation duration
AN	Anorexia nervosa
AOI	Area of Interest
BN	Bulimia nervosa
EAT-26	Eating Attitudes Test-26
ED/EDs	Eating disorder/Eating disorders
FC	Fixation count
FFD	First fixation duration
